# Measurements and predictions of diffusible hydrogen escape and absorption in catholically charged 316LN austenitic stainless steel

**DOI:** 10.1038/s41598-023-37371-y

**Published:** 2023-06-29

**Authors:** Melissa Weihrauch, Maulik Patel, Eann A. Patterson

**Affiliations:** School of Engineering, University of Liverpool, The Quadrangle, Brownlow Hill, Liverpool, L69 3GH UK

**Keywords:** Electrochemistry, Techniques and instrumentation, Metals and alloys

## Abstract

Hydrogen can have an impact on the service life of safety critical components, such as coolant pipes in nuclear reactors, where it may interact with other factors including irradiation. Hence, it is important to characterise such behaviour which in turn requires the capability to charge representative material specimens with hydrogen and to quantity the levels of hydrogen present. Hydrogen concentrations resulting from cathodic charging of 316LN stainless steel over short time periods (< 2 h) were estimated from hydrogen release rates obtained from potentiostatic discharge measurements and used to calibrate simulations based on Fick’s second law of diffusion in order to predict the hydrogen concentration after 24 h of charging. Leave-one-out cross-validation was used to establish confidence in results which were also validated using measurements from the melt extraction technique. The success of Fick’s second law for estimating escape rates showed that a majority of the absorbed hydrogen was diffusible rather than trapped. These results confirmed that the potentiostatic discharge technique can be used on materials with low diffusivity, and provide a new method through which hydrogen concentrations within a sample can be estimated after cathodic charging non-destructively without the need to remove samples from solution.

## Introduction

The presence of hydrogen is known to cause embrittlement and reduce service lifetimes of steel components. While austenitic stainless steels are generally thought to be less susceptible to the effects of hydrogen, due to their low hydrogen diffusivities, some past studies have shown that hydrogen can also reduce their fatigue life^1^.

In safety critical or extreme environments, uncertainty about the effects of hydrogen can make service lifetime predictions challenging. For example, in nuclear reactor coolant pipes, hydrogen may originate from radiolysis products and from deliberate modification of the coolant water chemistry by the addition of hydrogen. The added excess hydrogen is intended to react with oxygen molecules within the water, thereby preventing corrosion^2^. Additionally, in aqueous environments, hydrogen can form by the reduction of water. Water reduction can occur at cathodically protected components^3^ or at propagating cracks through passivation of newly formed surfaces^4^, hydrogen enters the material at the newly formed surfaces created by the propagation of a crack. Multiple studies have shown that hydrogen likely affects the crack propagation mechanisms of stainless steels in the coolant pipes in pressurised water reactors (PWR)^5,6^. The typical hydrogen concentration in as-received austenitic steels has been found to range between 2.2 and 3.4 wppm^7^.

Hence, studying the effect of hydrogen on the mechanical properties of steel is an important undertaking and requires a method to reliably incorporate known amounts hydrogen into the material. This paper focuses on the application of the potentiostatic discharge technique to measure absorbed hydrogen in 316LN steel. This grade of steel was studied due to its common use in the nuclear industry in components such as coolant pipes where hydrogen embrittlement is of concern. Thermal desorption spectroscopy (TDS) and melt extraction are well established techniques to measure hydrogen in steel. While these techniques are able to accurately measure hydrogen, and in the case of TDS trapping sites and energies can be identified, the potentiostatic discharge technique can be used in situ without the need to transfer the samples to separate machinery. To the best of the authors knowledge, the potentiostatic discharge method has not been explored for the austenitic phase, including 316LN steel. Experimental data were used to calibrate predictions of the escape rate made using Fick’s second law for relative short charging times. A linear relationship between the calibration factor and charge time was observed and used for predictions over longer time periods which were validated successfully using results from melt extraction measurements. Results obtained from this technique will enable future studies to understand the synergistic effect of hydrogen and radiation on the fatigue properties of austenitic steel which was a prime motivation for this study.

The next section provides a brief overview of methods of hydrogen charging and hydrogen concentration measurement techniques. The specimens, hydrogen charging apparatus, and concentration measurement techniques used in this study are described in the experimental methods. The simulation process is described in a separate section prior to the results, discussion and conclusions.

## Background

### Hydrogen charging methods

The commonly used methods to introduce hydrogen into steel are via cathodic or gaseous charging^8,9^. During gaseous charging, samples are placed in a pressure vessel filled with H_2_ gas, usually at high temperatures. For cathodic charging, samples are placed in an electrolyte and cathodically polarised, initiating a hydrogen evolution reaction at the sample surface. During electrolysis, variables including temperature, electrolyte, charging currents and charging time amongst other things affect the final hydrogen concentration. The large number of variables that must be controlled results in a wide range of hydrogen concentration measured by different authors. Hydrogen concentrations obtained in austenitic steels from gaseous and cathodic charging, have been summarized in Table 1. Sample thicknesses are provided to make comparisons between non-uniformly charged samples easier. Additionally, the depth of hydrogen diffusion for each given condition was estimated from Fick’s second law. Instances in which the entire sample was uniformly saturated with hydrogen have been specified.

**Table 1:** Variation in final hydrogen concentration in various austenitic stainless-steel grades obtained from gaseous and cathodic charging under various conditions of time, pressure, temperature and electrolyte used, based on the data from the literature^8,9^.

Steel grade	Charging condition	Temperature (°C)	Time (h)	Hydrogen concentration measurement method	Sample thickness (mm)	Hydrogen diffusion depth (mm)	Concentration (wppm)	Ref
316L	98 MPa	250	72	Thermal desorption spectroscopy (TDS)	0.77	0.77 (saturated)	~ 90	^8^
316L	98 MPa	85	1000			0.77	~ 35	
304	98 MPa	85	1000			0.77	~ 35	
304	98 MPa	250	72			0.77 (saturated)	~ 110	
316L	1 mA/cm^2^ 3% NaCl 3 g/L NH_4_SCN	RT	96			0.04	~ 7.5	
304	1 mA/cm^2^ 3% NaCl 3 g/L NH_4_SCN	RT	96			0.04	~ 7.5	
316L	50 mA/cm^2^ 0.1 M NaOH	95	72	Melt extraction	0.5	0.5	28–40^a^	^9^
316L	100 mA/cm^2^ 0.1 M NaOH		144			0.5	95–135^a^	
316L	50 mA/cm^2^ 0.5 M H_2_SO_4_		50			0.2	54–61^a^	

^a^Large variation in data due to different grain sizes being studied.

Table 1 demonstrates that charging temperature is an important variable effecting hydrogen concentration. Diffusivity increases exponentially with temperature, making it a key parameter that effects the rate and depth of hydrogen entry into materials. Hence, at high temperatures, uniform hydrogen concentrations can be achieved in relatively short timescales. The first two rows of Table 1 show the impact temperature has on absorbed hydrogen. The 316L specimens were both exposed to 98 MPa of hydrogen at temperatures of 85 °C or 250 °C respectively. Even though the charging time of the lower temperature (85 °C) specimen was increased from 72 to 1000 h, i.e. by an order of magnitude, the final hydrogen concentration reduced from 90 to 35 wppm. This can be attributed to reduced hydrogen solubility within the steel at lower temperatures. Moreover, cathodic charging at 95 °C resulted in similar hydrogen concentrations as the low temperature gaseous charged. As a comparison, room temperature experiments absorbed concentrations of only 7.5 wppm of hydrogen.

In Table 1, the 316L specimen charged at 100 mA/cm^2^ for 144 h showed similar hydrogen concentrations as gaseous charged specimens at 250 °C. This reflects the high hydrogen pressures (or fugacities) that can be achieved at the specimen surface during electrolytic charging using a high current. For gaseous hydrogen, concentrations within the material can be estimated using Fick’s second law of diffusion because the charging fugacity is known. This is not possible with electrolytic charging, hence predicting a final concentration is challenging.

Charging pressure and current density are the driving forces that affect the hydrogen concentration at the sample surface and therefore can be considered to be conceptually the same. In the past, equivalent hydrogen fugacities (i.e. pressures) have been calculated to allow comparison between the two methods of charging^10–12^. For instance, an investigation by Liu et al*.* found equivalent hydrogen pressures of up to 130 MPa during cathodic charging of 980DP steel with an overpotential of − 0.857 V^11^. Hence, large hydrogen concentrations could be obtained near the sample surface. During gaseous charging, steel can become prone to hydrogen induced cracking and blistering, especially in sulphur rich environments^13^. Similarly, high current densities can trigger austenite to martensite phase transformations and crack formation at the surface of 304 steel^14^. It is therefore important to ensure current density is kept sufficiently low to minimise changes to the material. Susceptibility to cracking during hydrogen charging appears to be related to the rate of hydrogen ingress, which can vary substantially between gaseous and cathodic charging making comparisons between the two method chalanging^13^.

Table 2 compares the absorbed hydrogen for different phases of steel charged at room temperature. At these temperatures, austenite has a diffusivity almost a million times smaller than martensite. Hence, steels containing ferritic and martensitic phases showed a concentration increase relatively quickly (≤ 24 h)^15,16^ whereas the 300 range of austenitic steels require comparatively long cathodic charging times to produce significant hydrogen concentrations. For instance, a concentration of 6.21 wppm was achieved in duplex steels after only 24 h of charging^15^. To achieve a similar level of hydrogen (7.5 wppm) in austenitic steels required a charging time of 96 h^8^. Achieving significant levels of hydrogen throughout the sample thickness in austenitic steels is therefore time consuming.

**Table 2:** Hydrogen concentration in austenitic, martensitic, ferritic, bainitic and duplex steels charged at room temperature, based on data from the literature^8,14,15^.

Steel grade	Phase	Charging condition	Time (h)	Measurement method	Sample thickness (mm)	Concentration (wppm)	Ref
SUS329J4L	Duplex (ferrite/austenite)	10 mA/cm^2^ 2.5pH sulfuric acid 0.1% by mass NH_4_SCN	24	TDS	1	6.21	^15^
Medium carbon steel	Martensitic with some bainite	− 1 V vs SCE 3% NaCl with 0.1% NH_4_SCN	3.4	TDS	1	1.45	^16^
316L	Austenitic	1 mA/cm^2^ 3%NaCl 3 g/L NH_4_SCN	96	TDS	0.77	~ 7.5	^8^

### Measurement of hydrogen concentration

Multiple techniques have been developed to measure hydrogen concentrations quantitatively and qualitatively. Techniques that provide spatial distributions of hydrogen concentration include nuclear reaction analysis (NRA) and energy recoil detection analysis (ERDA), which can also provide quantitative hydrogen concentration measurements as a function of depth^17^. The spatial resolution of ERDA is between 2 and 3 µm^18^. The hydrogen microprinting or silver decorating technique is a qualitative tool that has been used to visualise the diffusion behaviour of hydrogen in steels along grain boundaries and dislocations^19,20^. Kelvin probe microscopy can quantify hydrogen absorption with a high spatial resolution^21,22^. Finally, time-of-flight-secondary ion mass spectrometry (ToF–SIMS) has been utilised to qualitative and quantitively describe hydrogen diffusion behaviour in materials with spatial resolutions^23,24^. Lateral resolutions of 0.1 µm have been obtained using ToF–SIMS^25^. The technique has been used to observe the movement of deuterium from low to high stress regions in 304L stainless steels^23^. Similarly, the hydrogen distribution around a fatigue crack has been measured with SIMS^24^.

A wide range of techniques have also been developed to measure bulk hydrogen content. For instance, thermal desorption spectroscopy is often used to measure hydrogen content and the binding energy of trapping sites by observing desorbed molecules released from the metal; hydrogen is released from weak traps at low temperatures and strong trapping sites at high temperatures^26^. More detail on thermal desorption spectroscopy can be found in reference^27^. A similar technique for measuring hydrogen in samples is melt extraction, in which the sample is melted and released hydrogen is removed from the sample by a carrier gas. The change in thermal conductivity of the carrier gas is measured to determine hydrogen concentrations^28^. However, no differentiation between trapped and diffusible hydrogen can be made. A detailed explanation of the operating principles of melt extraction can be found in reference^29^.

The techniques mentioned so far are either destructive, resulting in all of the hydrogen in the sample being lost, or time consuming. While these methods are useful when sister samples are accessible, they are less applicable when only a small set of samples are available for testing. Other common non-destructive techniques, such as the Devanathan cell^30^, are not feasible at realistic time scales for thick samples with low diffusivities. However, some alternative methods exist, including low frequency impedance measurements^31^, and potentiostatic discharge^32^. Unlike potentiostatic discharge, low frequency impedance measurements cannot be done immediately after hydrogen charging. More recently, hydrogen has been measured in-situ during both gaseous and cathodic charging via the permeation technique, in a setup similar to the Devanathan cell method^33^. Like in the Devanathan cell, hydrogen diffuses from the charged to the non-charged surface which is polarised, and the resulting current measured, hence long timescales are needed in thick samples with low diffusivities. The potentiostatic discharge technique was chosen for this study due to its ease of implementation and ability to measure hydrogen release directly after charging without modifying the experimental setup.

### Potentiostatic discharge method

This study exploits the potentiostatic discharge technique, described by Yan and Weng^32^, where an oxidation potential is applied to the sample immediately after hydrogen charging and the resulting current is measured. The difference in current relative to a non-hydrogen charged sample can then be used to derive the hydrogen concentration. Ozdirik et al*.* verified concentrations obtained from potentiostatic discharge measurements by using melt extraction as a bench mark^34^. The results of their study have been summarized in Table 3. The difference in hydrogen content between electrolytic and melt extraction measurements was attributed to hydrogen escaping as samples were transferred to the melt extraction machine. From the data, plain carbon and steel grade As-Q showed the largest discrepancy, which were also the samples with the highest diffusion coefficient, and thus likely to lose the most hydrogen during transport for melt extraction.

**Table 3:** Hydrogen concentrations measured with the potentiostatic discharge method and melt extraction at room temperature (from Ozdirik et al.^34^).

Steel Grade	Phase	Charging condition	Time (h)	Method	Sample thickness (mm)	Concentration (wppm)	Diffusion coefficient (m^2^/s)	Ref
Plain carbon	Ferrite/pearlite	− 1.25 V (vs. Ag/AgCl) 1 M NaOH 8 g/L thiourea	4	Melt extraction	0.5	0.05	4.9 × 10^–10^	^34^
Plain carbon	Ferrite/pearlite			Potentiostatic discharge	0.5	0.41		
DP600	Ferrite/martensite			Melt extraction	0.9	0.07	6.9 × 10^–11^	
DP600	Ferrite/martensite			Potentiostatic discharge	0.9	0.08		
As-Q	Ferrite/martensite			Potentiostatic discharge	0.9	0.06	6.4 × 10^–10^	
As-Q	Ferrite/martensite			Potentiostatic discharge	0.9	0.02		

Compared to the other steels in Table 3, the diffusion coefficient of 316LN steel is almost a million times smaller. Therefore, the viability of the potentiostatic discharge technique, with low hydrogen diffusivity stainless steels needs to be confirmed. In this work, the potentiostatic discharge technique was applied to 316LN stainless steel and hydrogen escape rates were simulated using Fick’s second law of diffusion and compared to experimental results. By combining experimental and simulated data, total absorbed hydrogen was estimated and verified separately via melt extraction experiments.

## Experimental methods

### Specimen design and preparation

**Figure 1:**
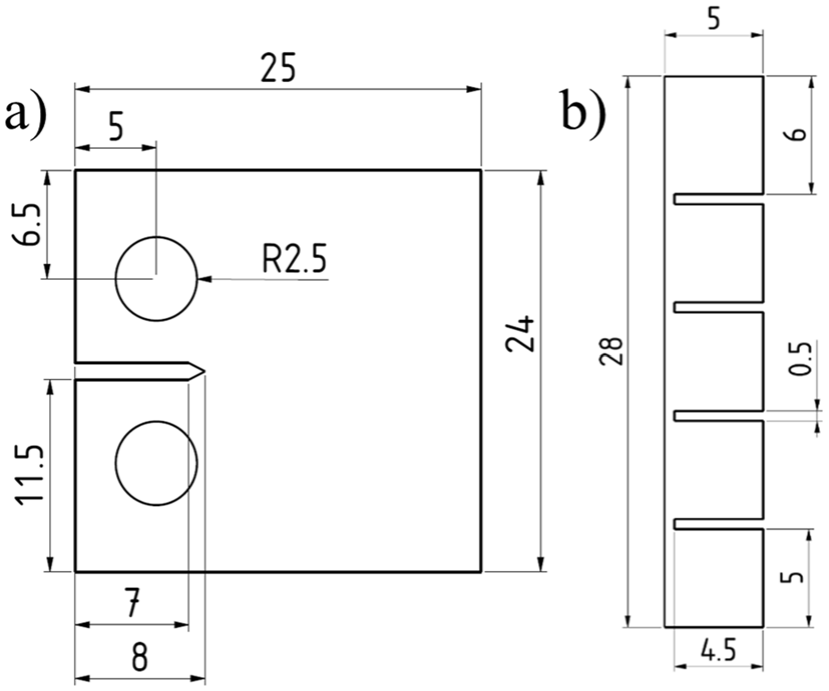
Specimen geometries for (**a**) potentiostatic discharge experiments and (**b**) melt extraction measurements. All dimensions are given in mm and the thickness of both specimens is 1.1 mm.

Compact tension specimens were used because the specimens were to be tested in fatigue in a separate set of experiments. The dimensions of the specimen, depicted in Fig. 1a, were chosen to be suitable both for fatigue tests and for ion irradiation studies in the future. Only the surface with the mirror finish was hydrogen charged. The other side was separated from the solution by applying in non-conductive Lacomit varnish procured from Agar Scientific (Agar Scientific Ltd, Stansted, UK). This resulted in an exposed surface area of 6.69 cm^2^. The 1.1 mm thick compact tension specimens were hydrogen charged for between 30 s and 2 h, as described below.

### Hydrogen charging

A 150 mm diameter billet of forged nuclear grade 316LN grade stainless steel procured from Pro-Roll (Pro-Roll Ltd, Sheffield, UK) was used to manufacture all of the specimens. Samples were metallographically prepared using a Buehler automated polisher (AutoMet™ 250 Grinder-Polisher, Buehler, Lake Bluff, IL). Samples were ground on both sides with SiC abrasive paper moving from grit size P400 to P800. They were subsequently polished with nine to one-micron diamond paste and given a final finish using colloidal silica on one side. The native oxide layer was kept intact, however cathodic hydrogen charging was expected to cause some degradation of the passive film^35^.

**Figure 2:**
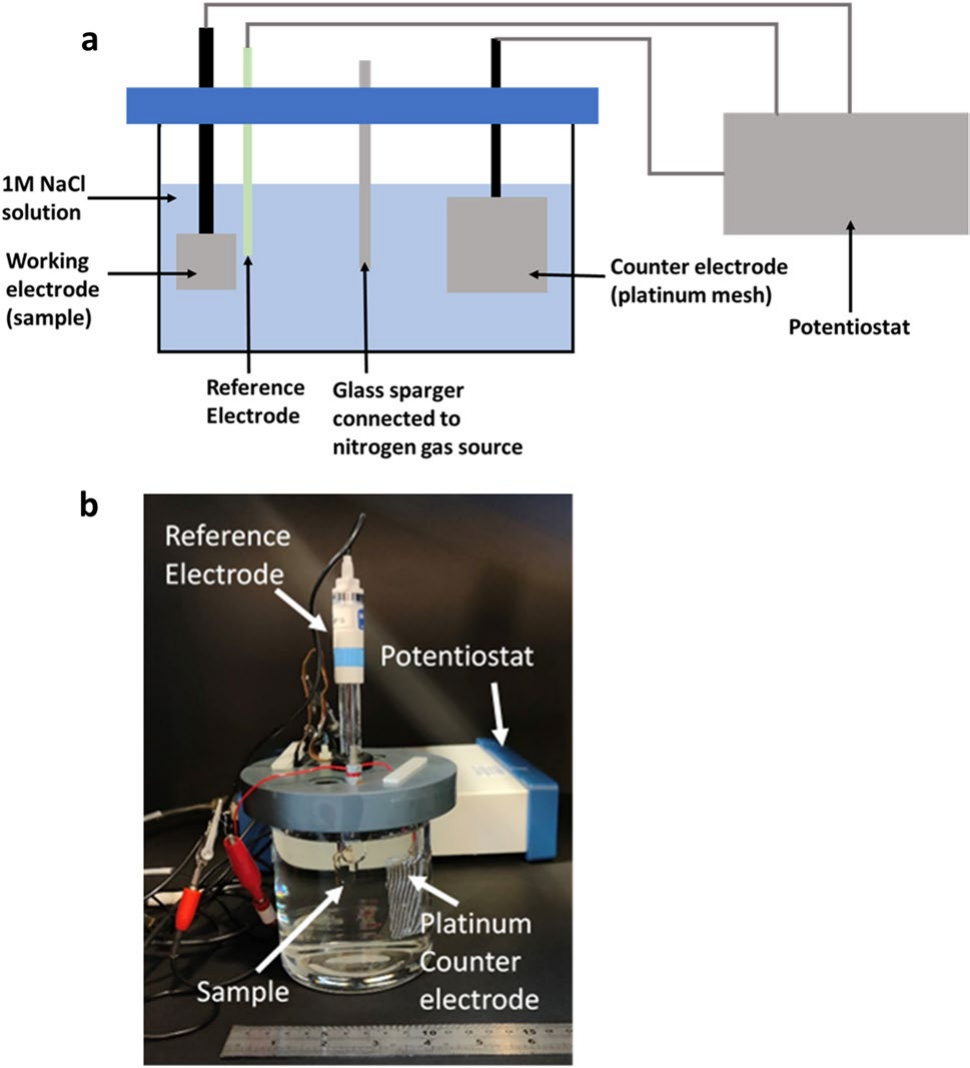
(**a**) Schematic of electrolysis cell setup, (**b**) picture of electrolysis setup for potentiostatic discharge experiments.

An electrochemical cell was custom-made to cathodically charge samples with hydrogen. Figure 2 shows a schematic and photograph of the setup developed for the potentiostatic discharge experiments**.** The hydrogen charging cell consisted of three main components: the working electrode (the sample), a counter electrode and a reference electrode.

A platinum mesh was used as a counter electrode, to complete the circuit of the electrochemical cell. The high stability of platinum meant that no corrosion reactions were taking place, while the large surface area provided by the mesh ensured that reaction kinetics were not limited.

A saturated calomel electrode was used as a reference electrode to measure the potential difference between the counter and working electrode. The reference electrode has a stable and well-defined potential, allowing it to provide a benchmark against which other potentials could be measured. The reference electrode and working electrode were placed within three millimetres of each other to give accurate measurements of the reactions occurring on the specimen surface. All three components were connected to a potentiostat (Model No.1010E, Gamry Instruments, Warminster PA) which was used to apply and measure the currents and potentials. Specimens were cathodically hydrogen charged at a current density of − 3 mA cm^−2^, which was calculated based on the surface area of the specimen that was exposed to the electrolyte. A small current density was chosen to mitigate surface modifications, such as transitions from austenite to martensite and surface cracking. Experiments on 316L steel at similar current densities have been performed in the past, and very small changes (if any) in martensite phase fraction were observed^7^.

1 M NaCl solution was used as an electrolyte and prepared using deionised water. Before the experiments, the glass container was washed with dish-washing soap and water, rinsed with deionised water and finally rinsed with isopropanol. Before starting potentiostatic discharge experiments, dissolved oxygen in the solution was removed by bubbling nitrogen through it by connecting a nitrogen gas source via a gas sparger which was placed in the solution. Previous studies have shown that at flow rates of around 25 mL/s, the removable oxygen limit is reached within an hour of bubbling gas through 1 L of solution^36^. During preliminary investigations with this setup, it was observed that the sparger became clogged with salt from the solution, reducing the gas flow rate; hence, the deoxygenation time was increased to 3 h to ensure the limit of removable oxygen was achieved.

### Potentiostatic discharge measurements

The potentiostat was programmed to apply a potential of − 0.2 V referenced to a standard calomel electrode (i.e. − 0.2 V vs. SCE) as soon as hydrogen charging was completed. The resulting current, attributed to the oxidation of hydrogen leaving the specimen, was measured. For low charging times (up to 15 min), data was collected for one-minute and negative currents were measured after this period which indicated that oxidation reactions had stopped. For longer charging times, the current was recorded for 2 min and measured currents became negative within this time frame.

The same specimen was reused for all tests. It is not expected that previous hydrogen charges influenced subsequent measurements as the recorded current became negative after every test. This indicated that all diffusible hydrogen had escaped. Additionally, experiments for some charge times were repeated and comparable results were obtained.

**Figure 3:**
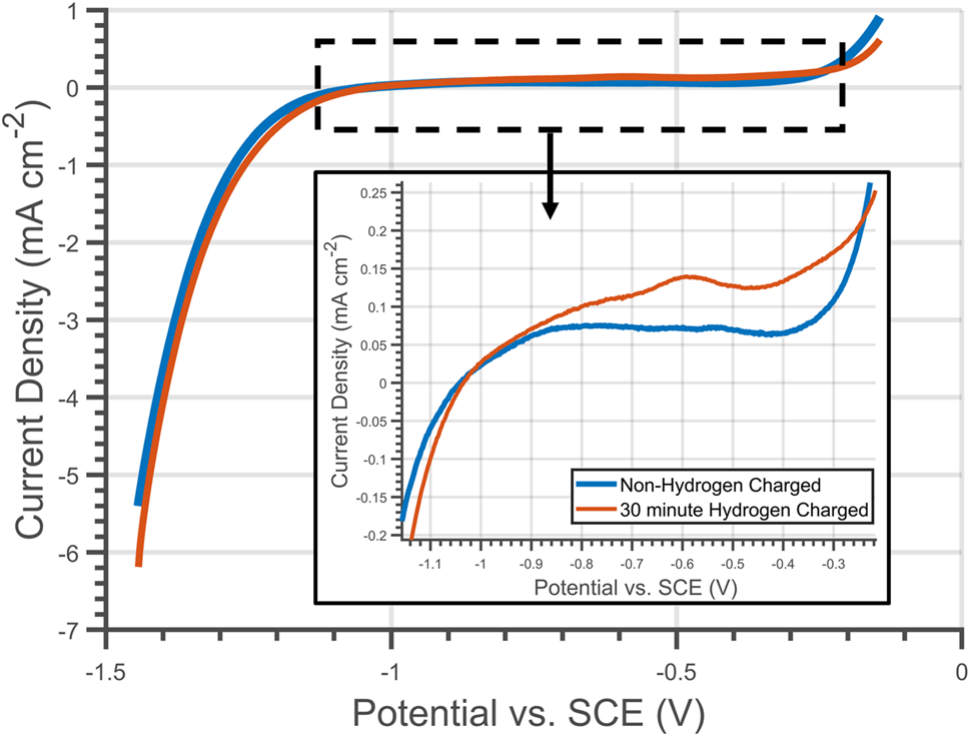
Forward sweep portion of cyclic voltammogram of 316LN stainless steel before (blue) and after (orange) a 30-min hydrogen charge.

A point of concern was that the oxidation of the steel could contribute to the measured anodic current. This would lead to unwanted surface modifications and skewed results. For instance, the formation of a passive layer could alter hydrogen escape rates. The potential was selected by considering cyclic voltammograms of hydrogen charged specimens measured during preliminary investigations. During cyclic voltammetry, a potential is cycled between two limits, and as the potential moves from one limit to the other, the current is measured. In the present case, cyclic voltammograms were recorded between potential limits of − 1.4 and − 0.1 V vs. SCE at a scan rate of 10 mVs^−1^ before and after hydrogen charging the specimen. Figure 3 shows the forward sweep of the cyclic voltammograms for the specimen before and after a 30-min hydrogen charge. After hydrogen charging a peak was produced at around − 0.59 V vs. SCE, which was attributed to the oxidation of hydrogen. The peak indicated that the oxidation of hydrogen commenced from this potential onwards. Hence, − 0.2 V vs. SCE was selected for potentiostatic discharge experiments as a suitable potential for hydrogen oxidation. This value was deemed large enough for oxidation of hydrogen atoms to occur, but low enough to prevent oxidation of the steel surface. The non-hydrogen charged curve in the cyclic voltammogram shows that, at this potential, the current density was low, limiting other reactions not associated with the oxidation of hydrogen.

The temperature and pH were recorded prior to and after the potentiostatic discharge experiments with an electronic thermometer and litmus paper. No observable changes in pH were recorded while the temperature increased from 20 to 32 °C during hydrogen charging. The temperature increase was greatest for longer charging times, while it was less for shorter charging times.

### Melt extraction measurements

Hydrogen concentrations in specimens were measured via melt extraction to allow comparison to electrochemical measurements. Results from the potentiostatic discharge method gave an estimate of absorbed hydrogen instead of total hydrogen concentration. To compare results from the two methods, concentration differences were calculated from melt extraction measurements by finding the difference between hydrogen in charged and pristine specimens.

Two types of smaller specimens were designed to fit the specifications of the melt extractor (ELTRA OH-900, ELTRA, Haan, Germany). Pristine specimens were 5 × 5 × 1.1 mm in size. Specimens for hydrogen charging were manufactured according to the dimensions given in Fig. 2b with a thickness of 1.1 mm**.** The specimen design had four 5 × 5 mm sub-sections which could be easily separated after charging, effectively allowing four specimens to be charged simultaneously. These specimens were polished to a mirror finish on both faces, allowing hydrogen charging from all surfaces. In total eleven pristine specimens and three hydrogen charged specimens (i.e. twelve specimens in total) were measured with melt extraction.

As melt extraction measured bulk concentration, hydrogen charging times had to be increased sufficiently to detect a change in hydrogen content. No significant changes from the short charges described in the potentiostatic measurement section would have been measured. Specimens were hydrogen charged for 24 h in a corrosion cell with a setup similar to that described previously. Instead of a potentiostat, a power supply was used (Model E36105A, Keysight, Santa Rosa, CA). As potentials did not have to be measured, a reference electrode was not used. All specimens were weighed and cleaned with isopropanol prior to analysis. Typically, no more that 15 min passed between hydrogen charging and measurement. The parameters used for the measurements are listed in Table 4.

**Table 4:** Parameters used for melt extraction measurements with the ELTRA OH-900.

Parameter	Value
Outgassing time	30 s
Outgassing power	4.5 kW
Purge time	10 s
Stability time	30 s
Integration delay	17 s

## Measuring and simulating hydrogen escape rates

Hydrogen escape rates were obtained with the potentiostatic discharge technique via the measurement of the hydrogen oxidation current. The experimental results were compared to simulations based on Fick’s second law, to compare deviations in experimental and theoretical results. As Fick’s second law only considers diffusible hydrogen, conclusions about trapping behaviour can be made.

In the investigation of Yan et al*.*, measured anodic currents in potentiostatic discharge experiments were attributed to the oxidation of adsorbed hydrogen^32^. The anodic current encourages the formation of a hydrogen ion [reaction (1)] instead of the production of molecular hydrogen [reaction (2)].

1 $$ H_{ads} \to H^{ + } + e^{ - } $$

2 $$ 2H_{ads} \to H_{2} $$

The associated change in current caused by reaction (1) can be equated to the hydrogen release rate as follows:

3 $$\frac{dH}{dt}=\frac{{I}_{measured}-{I}_{background} }{{q}_{e}}$$

where *H* is the number of hydrogen atoms, *t* is time, *I* is current and, ${q}_{e}$ is the elementary charge (1.602 × 10^–19^ Coulombs). The total hydrogen released can be experimentally evaluated by integrating the change in current with time:

4 $${H}_{Total}=\frac{\int \left({I}_{measured}-{I}_{background}\right)dt}{{q}_{e}}=\frac{Q}{{q}_{e}}$$

where *Q* is charge. Measured currents were integrated from *t* = 0 up to the time that measured currents become negative (indicating that hydrogen oxidation had stopped). It should be noted that the amount of hydrogen is quantified in terms of the number atoms, and not as a unit of concentration. In systems with few traps, the total released hydrogen should be roughly equal to the total absorbed hydrogen. Therefore, the calculated total of escaped hydrogen provides an estimate of the total absorbed hydrogen.

To compare the measured hydrogen release rates to Fick’s second law, hydrogen escape rates were estimated from an expression based on Fick’s second law of diffusion and given by Yagodzinskyy et al.^37^:

5 $$C\left(x\right)= \frac{4{C}_{0}}{\pi }\sum_{n=0}^{\infty }\frac{(-{1)}^{n}}{\left(2n+1\right)}\mathrm{cos}\left(\frac{\left(2n+1\right)\pi x}{h}\right)\left(1-{e}^{-\frac{{\pi }^{2}{\left(2n+1\right)}^{2}D\left({T}_{s}\right){t}_{s}}{{h}^{2}}}\right){e}^{-\frac{{\pi }^{2}{\left(2n+1\right)}^{2}D\left({T}_{d}\right){t}_{d}}{{h}^{2}}}$$

where ${C}_{0}$ is the hydrogen concentration on the surface, $h$ is thickness from the centre to the surface of the specimen, $D\left({T}_{s}\right)$ and $D\left({T}_{d}\right)$ are the diffusion coefficients at the hydrogen charging temperature and desorption temperature respectively, and ${t}_{s}$ and ${t}_{d}$ are hydrogen charging and desorption times. As the concentration at the surface was unknown, only the concentration fraction, $\frac{C\left(x\right)}{{C}_{0}}$, was computed. The variation in diffusion coefficient with temperature was accounted for using an Arrhenius relation^38^:

6 $$D={D}_{0}{e}^{\frac{-{Q}_{a}}{RT}}$$

where ${D}_{0}$ is the diffusion pre-exponential factor, ${Q}_{a}$ is the activation energy for hydrogen diffusion via interstitials (i.e. through the metal lattice), for which the units are J/mol. *R* is the gas constant and *T* is temperature in Kelvin. The parameters used for the simulations are summarised in Table 5.

**Table 5:** Parameters used to predict hydrogen desorption rates via Fick's second law.

Parameter	Value
Diffusion pre-exponential factor (${D}_{0}$)	8.9 × 10^–7^ m^2^s^−1^^39^
Activation energy (${Q}_{a}$)	53.9 × 10^3^ Jmol^−1^^39^
Temperature ($T$)	32 °C
Thickness ($h$)	1 × 10^–3^ m
Summation upper limit ($$n)$$	100,000
Depth upper limit ($x$_max_)	10 × 10^–6^ m

Hydrogen release rates for the first 2 min after cathodic hydrogen charging were computed for charge times ranging from 30 s to 2 h using a purpose-written MATLAB code.

## Results

### Potentiostatic discharge

**Figure 4:**
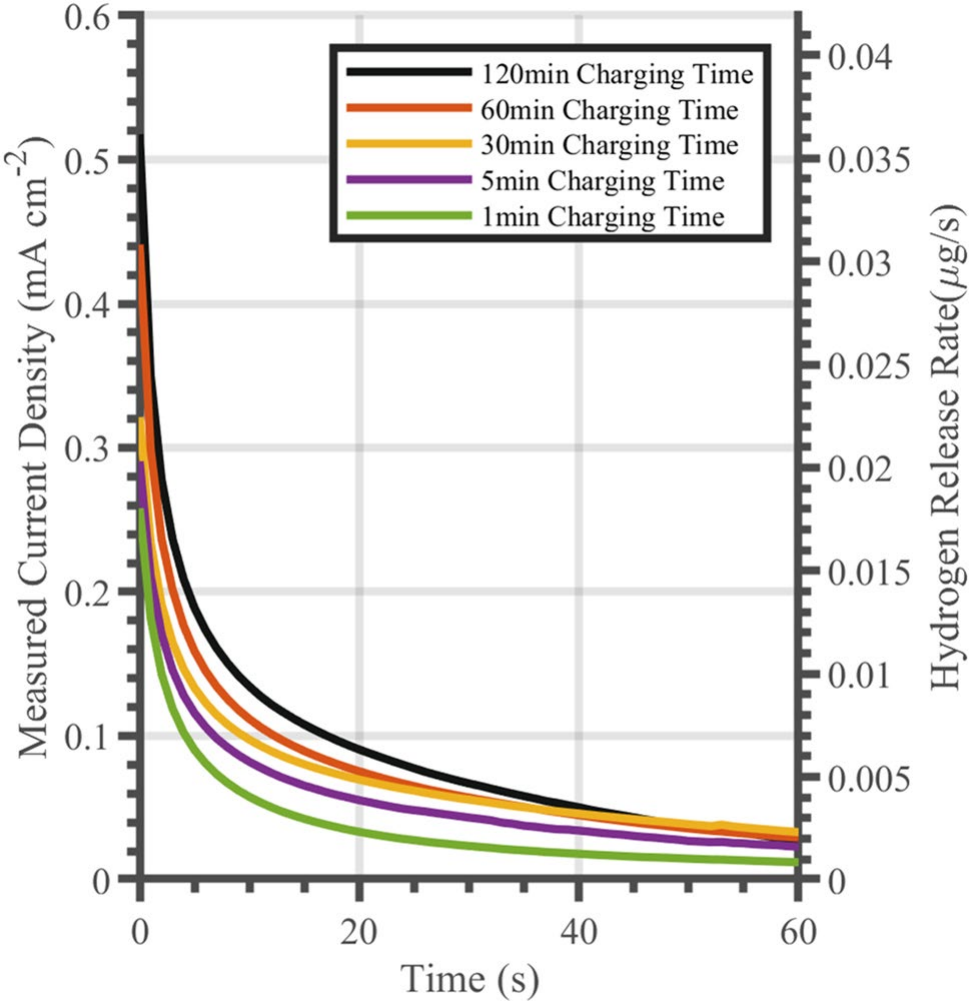
Measured potentiostatic discharge current density relative to a pristine specimen after hydrogen charging. On the x- axis time t = 0 represents the time from hydrogen charging. Each curve represents a different charge duration.

A decrease in current with desorption time can be seen in Fig. 4, note that time *t* = *0* represents the time from hydrogen charging being completed. The plotted currents are the difference in current compared to measurements from a non-hydrogen charged specimen. Currents were at their largest immediately after hydrogen charging and declined over time in an exponential manner. This is representative of hydrogen release being at its maximum immediately post-charging and reducing gradually as the hydrogen concentration decreased and approached zero. The current density at *t* = *0* increased with charge time, due to increased amounts of hydrogen being absorbed.

**Figure 5:**
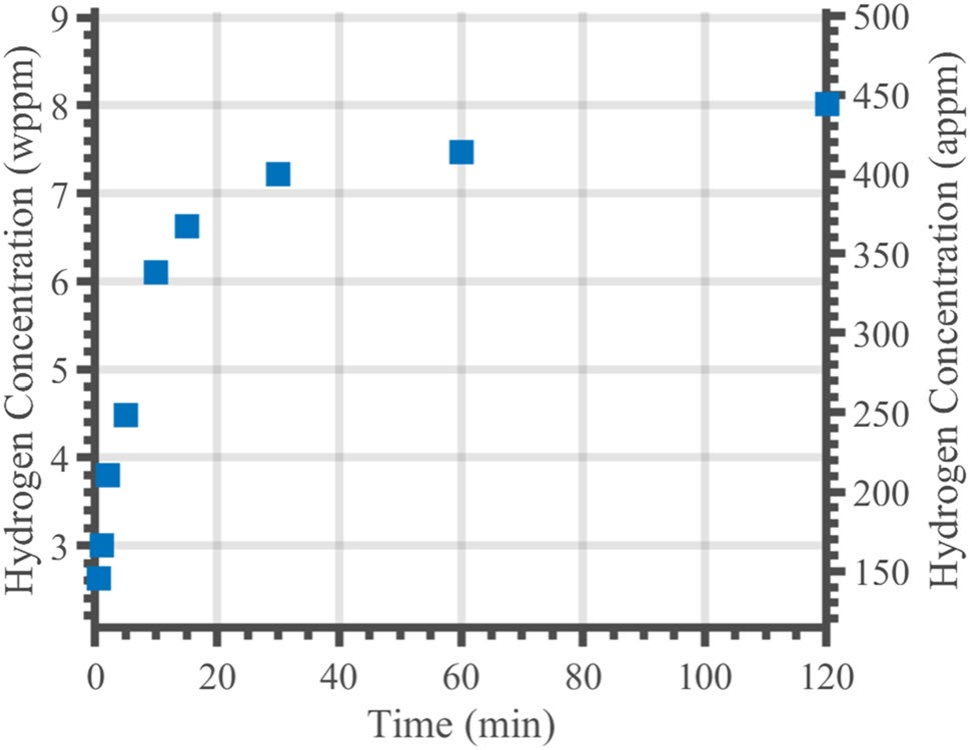
Hydrogen concentration changes obtained for different hydrogen charge times as estimated from potentiostatic discharge curves within the first 10 µm of the specimen.

Total absorbed hydrogen for each charge time was estimated by integrating the curves in Fig. 4 and applying Eqs. (3) and (4). Using Eq. (5) it was predicted that hydrogen would not have diffused beyond a depth of 10 µm within 120 min, hence the change in concentration was calculated only to a depth of 10 µm. Figure 5 shows the hydrogen concentrations within the first 10 microns as a function of charging time. Initially, a sharp rise in hydrogen concentration with charging time can be seen. After 20 min of charging, saturation takes place with a very slow accumulation of hydrogen, becoming more pronounced at longer charging times. This trend is in agreement with other studies that observed an upper limit of measured hydrogen content^34^.

### Simulations

The predicted hydrogen escape rate after 2 h of hydrogen charging was calculated using the purpose-written MATLAB code. As the concentration at the surface, ${C}_{0}$ was an unknown parameter, the simulations results were in arbitrary units and a calibration factor had to be applied to allow direct comparison between the measured and predicted results. The calibration factor was the ratio of the measured current density and predicted unitless release rates at *t* = 0.5 s during experiments:

7 $$Calibration \, Factor=\frac{Measured  \, Current  \, {Density}_{t=0.5 \, \text{s}}}{Simulation  \,R{esults}_{t=0.5 \, \text{s}} }$$

**Figure 6:**
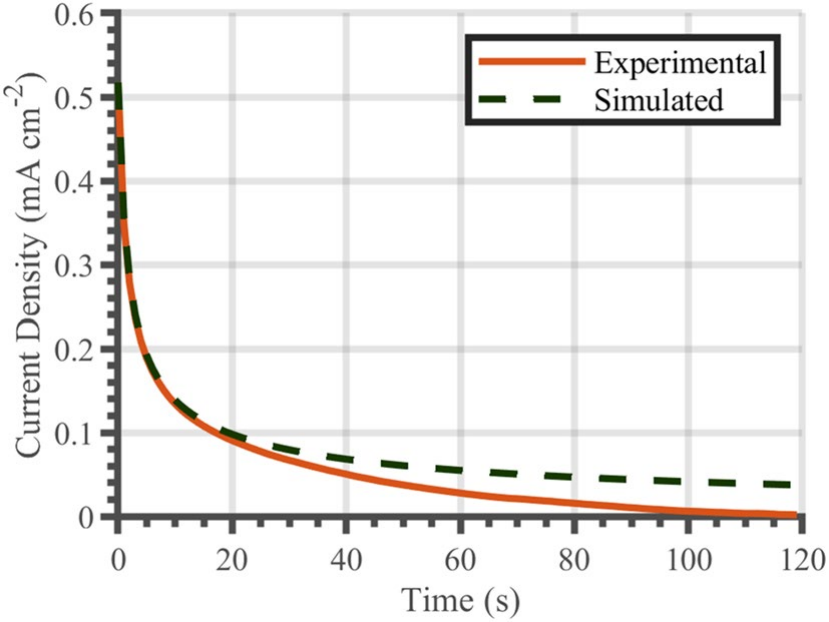
Oxidation current density after 120 min of hydrogen charging. The solid line represents experimental data. The dashed line shows the predicted hydrogen release rate from calibrated simulations based on Fick’s second law.

A comparison between the predicted and measured results for a 2-h charge is shown in Fig. 6. The predictions show the same exponential decline in hydrogen release rate that was observed experimentally. In the initial stages of release (up to* t* = 30 s), Fick’s second law was able to accurately estimate release rates. The first 30 s accounts for 66% of the released charge, meaning that this is a significant portion of the desorption process. At later times, the simulations and experimental results started diverging, with the simulation over-predicting the hydrogen release rates.

The discrepancy between experimental data and simulated predictions were further investigated by computing the difference between the predicted and experimental results for various charge times. The difference was always low during the initial stages of desorption. For shorter charge times of up to 30 min, they seem to approach zero as desorption time increased. On the other hand, for longer charging times, differences between the predictions and measurements increased with time.

**Figure 7:**
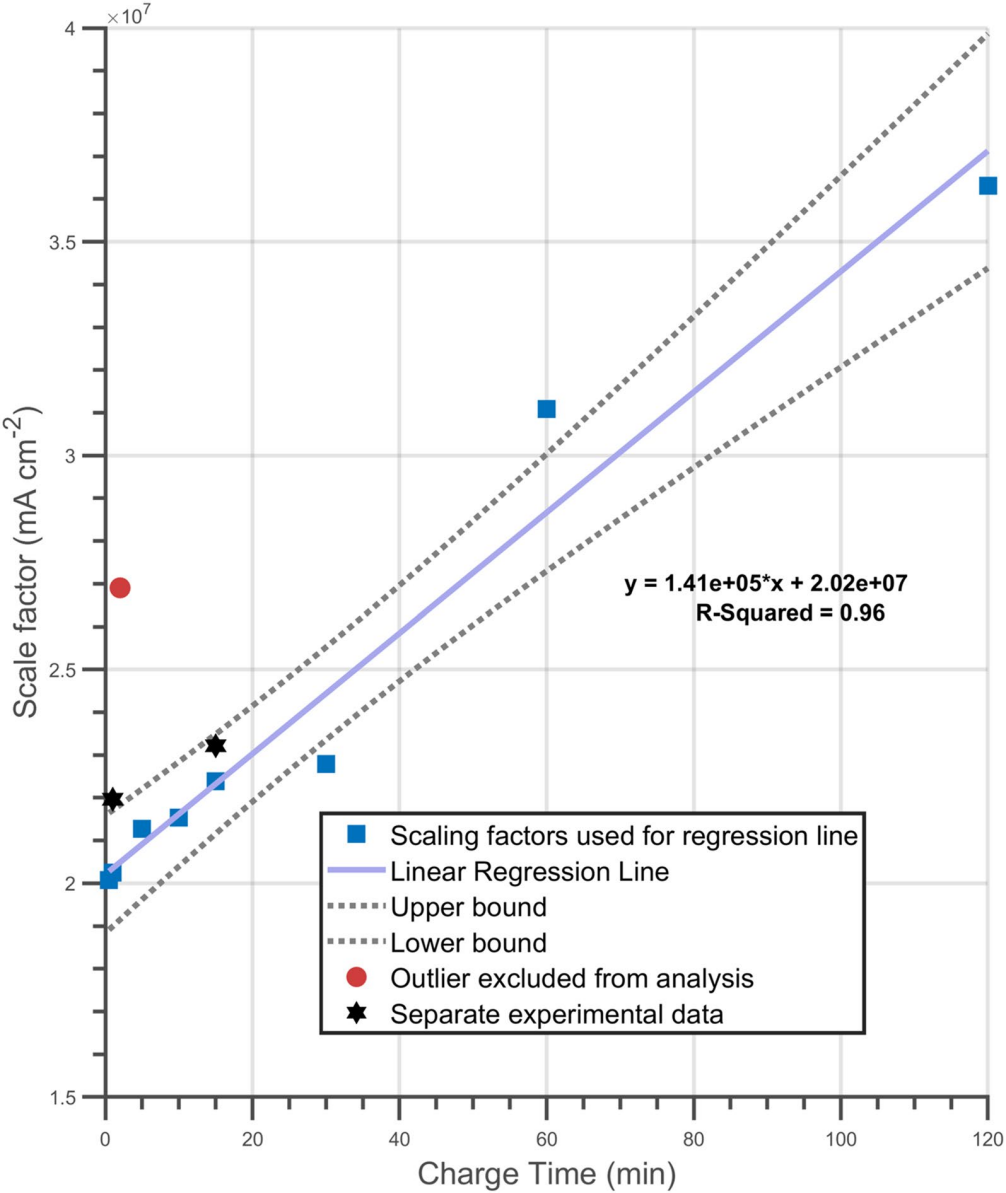
Calculated calibration factor for simulations predicting oxidation currents. Blue squares are data used to generate the line of best fit, the black squares represents two additional measurements to varify repeatability of data, the red square is an outlier.

The calibration factor increased with charge times, which is reflective of hydrogen release rates increasing proportionally with charge time (see Fig. 4). Figure 7 shows a linear relationship (solid line) between charge time and the calibration factor. The dashed lines represent the upper and lower bounds for the 95% confidence intervals of the linear regression, with the majority of the data points within these bounds.

The red circle represents an outlier that was not included in analysis. Further investigation of this point (representing a two-minute charge) showed that currents measured immediately after charging were greater than those observed after 5 min of charging. As mentioned earlier, hydrogen release rate at *t* = 0 should be proportional to charge time. Hence, it was concluded that a mistake had been made in the experimental work so it was decided to exclude this outlier from the regression analysis shown in Fig. 7.

The whole calibration process, excluded in the regression analysis, was repeated for two charging times and the results are shown in Fig. 7 as black hexagons which are within or near the calculated 95% confidence bounds, verifying the repeatability of the process.

To increase confidence and establish the validity of the linear relationship between charge time and calibration factor, Leave One Out Cross Validation (LOOCV) was utilised. Here, the R-squared value of the linear regression line was recalculated eight times (equal to the total number of data points), leaving out one data point each time and giving 8 separate R-squared values. An average R-squared value of 0.96 was obtained with a minimum of 0.93 and a maximum of 0.99. The high R-squared values shown by LOOCV further justify the exclusion of the outlier and the utilization of a linear fit.

### Hydrogen measurements with melt extraction

**Figure 8:**
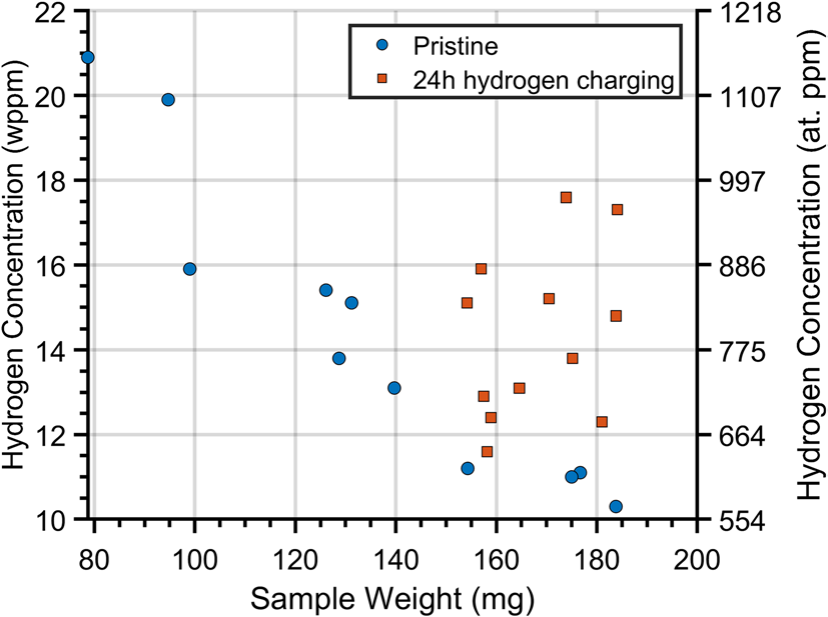
Hydrogen concentrations from melt extraction measurements of pristine non-hydrogen charged (circles) and 24 h hydrogen charged (squares) specimens. The data demonstrates a dependency of concentration on specimen weight.

Melt extraction was used to measure changes in hydrogen concentration for longer periods of charging time. In the pristine specimens, the hydrogen content was 14.3 wppm with a standard deviation of 3.6 wppm. As seen in Fig. 8, the high standard deviation can be attributed to the large range in weight of the uncharged specimens (79–183.8 mg). The figure shows an inverse relationship between specimen weight and hydrogen content. Therefore, only pristine specimens with similar weights to those of the charged specimens (154–184 mg) were included in the calculations, which resulted in a mean concentration of 10.9 wppm with a standard deviation of 0.41 wppm for the pristine samples. The final concentration after 24 h of charging was 14.3 wppm with a standard deviation of 2 wppm. The large weight range was due to varying amounts of material being removed during polishing. A similar trend has been observed by Hassel et al*.*^39^ with the ELTRA OH analyser, who found that measurements of hydrogen content varied with weight and shape; however, after discussion with the manufacturer, this problem was attributed to a calculation error in the machine software^40^.

The results from the melt extraction technique were compared with those from the simulation calibrated with the potential discharge technique. The average difference in concentration between pristine specimens and those hydrogen charged for 24 h was found to be 3.4 wppm with a standard deviation of 2.04.

## Discussion

Potentiostatic discharge experiments with a custom-made electrolysis apparatus have been performed on 316LN stainless steel and showed that hydrogen release rates can be measured by monitoring current changes. Concentration increases of over 8 wppm were obtained to a depth of 10 µm from the surface of the material after a 2-h charge. This depth is of importance as future work will consider the additional impact of ion irradiation defects which reside in a similar depth range.

While austenitic steels had not been studied with the potentiostatic discharge technique, the results from the experiments showed similar trends to those found previously in studies on martensitic and ferritic steels. For example, Ozdirik et al*.* found the hydrogen saturation behaviour with charging time in martensitic and ferritic steels^34^. The hydrogen saturation behaviour that Ozdirik et al*.* found was also observed in the austenitic steel studied here (see Fig. 5). The similarity in results and observed trends in the two studies demonstrates that the technique can be extended to austenitic steel to monitor hydrogen escape. Additionally, significant changes in hydrogen concentration were achieved in the near-surface region after short cathodic charging periods in both studies.

Hydrogen desorption occurred over much shorted time scales then the charging time, for instance in the 15 min hydrogen charge, negative currents were reached within 1 min of discharge. This was attributed to two effects. First the hydrogen desorption kinetics could have been accelerated by the oxidation current across the sample surface. Second, trapped hydrogen may play a role. Strongly trapped hydrogen would have remained in the sample, and therefore never reached the sample surface to be oxidised. On the other hand, weakly trapped hydrogen could have escaped through different mechanisms. For instance, it has been suggested that weakly trapped hydrogen in martensitic steel is released rapidly during degassing^34^.

A hydrogen concentration of 8wppm was measured after 2 h of charging within the first 10 µm of the sample (see Fig. 5), translating to 0.07 wppm across the entire sample thickness. This value is in line with the measurements given in Table 3, ranging from 0.02 to 0.41wppm depending on steel grade. The similarity in concentration between the austenitic steel used here and the other steel grades of different phases is unexpected due to the large discrepancies in diffusion coefficients. The comparatively high solubility^41,42^ of hydrogen in austenitic steel could explain the larger than expected concentration. While hydrogen moves through austenitic steel more slowly due to the low diffusivity, austenite has the potential to absorb more hydrogen due to the higher solubility. It is therefore possible that due to the short charging times involved, solubility instead of diffusivity was the dominant material property governing hydrogen absorption.

Another factor leading to the relatively large measurement of hydrogen are differences in experimental setup and procedure compared to other studies. For instance, in this work, charge was integrated over a longer time period of 2 min as compared to the fifty-second-time frame used by Ozdirik et al*.* Additionally, a higher oxidation potential was applied, which could have contributed to higher measured currents.

The simulations were in good agreement with the experimental data, see Fig. 6, especially for the first thirty-seconds of hydrogen charging. This shows that hydrogen release behaviour can be predicted with Fick’s second law of diffusion. The success of Fick’s second law for estimating escape rates implies that a majority of the absorbed hydrogen was diffusible rather than trapped. Hence, the effect of the introduction of additional trapping sites, via ion irradiation induced defects for instance, could be deduced from a deviation of the experimental measurements from the predictions.

The linear dependence found between the calibration factor, for the simulation, and the hydrogen charge times, is a significant observation. The relationship could be utilised in the future to calibrate predictions without the need to perform potentiostatic discharge experiments. Thus, hydrogen release and total absorbed hydrogen for longer charge times could be estimated without performing lengthy experiments. To test this hypothesis, hydrogen release over a 24-h period was predicted and compared with the hydrogen concentration measured via melt extraction. An increase in bulk hydrogen concentration between 0.9 and 1.3 wppm was predicted using the simulation calibrated with the potentiostatic discharge data. The procedure used to predict hydrogen concentrations from simulations can be found in the Supplementary information 1 and 2. Results from the melt extraction measurements showed a mean increase of concentration equal to 3.4 wppm with a standard deviation of 2.04 wppm. The upper bound of the predicted concentration increase (1.3 wppm) approximately within one standard deviation of the mean of the measurements from the melt extraction. Hence, it can be concluded that the simulation based on Fick’s second law using a calibration factor extrapolated from potentiostatic discharge tests can be used to estimate hydrogen ingress into steels for long charging times beyond the data used for calibration.

There are a number of sources of error, including the formation of molecular hydrogen during potentiostatic discharge measurements, which was not detectable and was unaccounted for. Likewise, hydrogen trapped at defects did not desorb, causing an underestimation of results. Work analysing thermal desorption spectroscopy peaks by Silverstein and Eliezer^43^ found three reversible trap types in 316L steel, namely trapping at: the elastic field around a dislocation, the dislocation core, and the martensitic phase boundary. The authors also noted the possibility of high angle grain boundaries contributing to the measured peaks. Hydrogen at such locations would not have diffused out of the specimen, and would therefore not have been measured. Studies have also suggested that some weakly trapped hydrogen can be released during anodic polarisation^44^. It should be highlighted that melt extraction measured all of the hydrogen within the samples, while potentiostatic discharge could only account for diffusible and weakly trapped hydrogen. Due to the large number of traps in austenitic steels, potentiostatic discharge is expected to underestimate the amount of total absorbed hydrogen.

Furthermore, trapping behaviour is not considered in Fick’s second law of diffusion, for which it was assumed that all absorbed hydrogen would be re-released. This led to an over prediction of hydrogen release rates. Additionally, cold working from polishing would have produced an increase in near surface dislocations, influencing the effective diffusivity near the surface. Experiments on 316LN have shown that tensile cold deformation increased the material’s susceptibility to hydrogen embrittlement due to an increased number of dislocations^45,46^. It is possible that strains induced by polishing may lead to a similar increase in surface dislocations. As low diffusion depths of under 15 µm are relevant here, the dislocations introduced from polishing could have affected diffusion and trapping behaviour. However, it is expected that trapped hydrogen accounted for a small fraction of absorbed hydrogen, as simulations predicted hydrogen desorption well.

During melt extraction the formation of a hydroxide layer on the steel may have led to an over reporting of hydrogen content. While some hydrogen would have escaped during the transfer time between hydrogen charging and measurements being performed, this has been considered to be negligible in past studies due to the material’s low diffusivity^7^.

To summarize, the results from this work demonstrated that potentiostatic discharge technique is a simple method to measure hydrogen desorption rate in 316LN steel immediately after cathodic charging. To the best of the authors knowledge these experiments had not been performed previously on austenitic steels with low diffusivity. The results can be used to calibrate simulations and to estimate the total absorbed hydrogen for long charge times, beyond those readily evaluated experimentally. This should enable future work to explore the hydrogen release during fatigue experiments post charging. An additional area of exploration could be the use of the potentiostatic discharge technique to identify changes in hydrogen release behaviour of irradiated specimens.

## Conclusions

Potentiostatic discharge experiments with a custom-made electrolysis setup on 316LN stainless steel showed that hydrogen release rates can be measured by monitoring current changes. Concentration increases of over 8 wppm could be obtained in the first 10 µm of the material after a 2-h charge.

Hydrogen release rates were predicted using Fick’s second law and calibrated using experimental data from the potentiostatic discharge technique. It was found that the early stages of hydrogen desorption, accounting for 66% of the total released hydrogen, could be predicted reliably. Additionally, the calibration factor varied linearly with hydrogen charging time. Using this relationship, the simulation was calibrated and used to predict absorbed hydrogen concentration after 24 h of charging to be between 0.9 and 1.3 wppm. These predictions were validated using independent measurements from the melt extraction technique which gave a mean concentration increase of 3.4 wppm with a standard deviation of 2.04 wppm after 24 h of charging. Thus, the predicted and measured results are approximately within one standard deviation of one other, providing a validation of the results from the simulation calibrated with the potentiostatic discharge measurements. Additionally, a baseline behaviour for hydrogen release in unirradiated specimens has been established which will be valuable in future studies on irradiated specimens.

## Supplementary Information

Supplementary Information 1.

Supplementary Information 2.

Supplementary Information 3.

## Data Availability

All data analysed during this study are included in this published article and its supplementary information files.
