# Supplementary material for: Measurements and predictions of diffusible hydrogen escape and absorption in catholically charged 316LN austenitic stainless steel
Source: Sci Rep. 2023 Jun 29;13:10545. doi: 10.1038/s41598-023-37371-y (PMC10310816; doi:10.1038/s41598-023-37371-y)
Supplement: Supplementary file 2 — Supplementary Information 2. [file 41598_2023_37371_MOESM2_ESM.pdf]

Measurements and Predictions of Diffusible Hydrogen Escape and Absorption in Catholically Charged 316LN Austenitic Stainless Steel

Melissa Weihrauch*, Maulik Patel, Eann A. Patterson

Supplementary file containing the procedure to predict hydrogen concentration from Fick's second law simulation

The simulation results of hydrogen escape rate after a twenty-four-hour hydrogen charge (titled '24h Simulation.txt') have been provided in the supplementary files. In the following example, hydrogen concentrations in a sample after twenty- fours of hydrogen charging at room temperature is estimated from simulation results:

Calibration of Simulation

1) Use linear relationship in figure 7 to obtain calibration factor:
   a. $Calibration\,factor\ =\ 1.41 \times 10^5 \times t + 2.02 \times 10^7$
      Where the units for calibration factor are (mA/cm^2) and t is charging time in minutes
   b. $Calibration\,factor = 1.41 \times 10^5 \times (24 * 60) + 2.02 \times 10^7$
      $= 2.23\ \times 10^8 \mathrm{mA/cm^2}$
2) Calibrate the simulated hydrogen escape rate using the calibration factor to obtain current density using the below equation
   a. $Predicted\ current\ density\ =\ Calibration\,factor\ \times$ $Simulated\ H\ escape\ rate$
   b. Where the units of current density are $\mathrm{mA/cm^2}$
3) Convert current density to current by multiplying by surface area (6.69cm^2)

Calculating Mass of Released Hydrogen

1) Calculate total number of released hydrogen atoms from:
   a. $H_{Total} = \frac{\int(I)dt}{q_e} = \frac{Q}{q_e}$
      Where I is current in Amperes, Q is charge, t is desorption time and q_e is the elementary charge (1.602×10^{-19} Coulombs).
   b. In this example
   c. $H_{Total} = \frac{\int(I)dt}{q_e} = \frac{0.393}{1.602 \times 10^{-19}} = 2.453\ \times 10^{18}$ atoms
2) Convert atoms of hydrogen to mass:
   a. $H_{mass} = \frac{H_{Total} \times M_H}{N_A} = \frac{2.453\ \times 10^{18} \times 1.01}{6.022 \times 10^{23}} = 4.114 \times 10^{-6}\mathrm{g}$

Calculating Hydrogen Concentration

1) Find concentration using:
   a. $C = \frac{H_{mass} \times 1 \times 10^6}{S_{mass}} = \frac{4.114 \times 10^{-6} \times 1 \times 10^6}{4.2} = 0.98$ wppm
   b. Where C is hydrogen concentration in wppm and S_{mass} is the mass of the sample in grams
